# A Novel Fluoroimmunoassay for Detecting Ruscogenin with Monoclonal Antibodies Conjugated with CdSe/ZnS Quantum Dots

**DOI:** 10.3390/molecules22081250

**Published:** 2017-07-26

**Authors:** Hongwei Zhang, Tao Xu, Lan Gao, Xiufeng Liu, Jihua Liu, Boyang Yu

**Affiliations:** 1 Jiangsu Provincial Key Laboratory for TCM Evaluation and Translational Development, China Pharmaceutical University, Nanjing 211198, China; zhwhhxx@sina.com (H.Z.); xt6111116@163.com (T.X.); 15298360058@163.com (L.G.); xf.liu@cpu.edu.cn (X.L.); 2 State Key Laboratory of Natural Medicines, China Pharmaceutical University, Nanjing 211198, China

**Keywords:** fluorescent immunosorbent assay, ruscogenin, monoclonal antibody, quantum dots, tissue distribution

## Abstract

Ruscogenin (RUS) is a steroidal sapogenin found in *Ruscus aculeatus* and *Ophiopogon japonicus* with several pharmacological activities. In the work reported herein, a novel method termed competitive fluorescence-linked immunosorbent assay (cFLISA) based on monoclonal antibodies (mAbs) coupled with quantum dots (QDs) was developed for the quick and sensitive determination of RUS in biological samples. The mAbs against RUS were conjugated with CdSe/ZnS QDs by the crossing-linking reagents and an indirect cFLISA method was developed. There was a good linear relationship between inhibition efficiency and logarithm concentration of RUS which was varied from 0.1 to 1000 ng/mL. The IC_50_ and limit of detection (LOD) were 9.59 ng/mL and 0.016 ng/mL respectively, which much lower than the enzyme-linked immunosorbent assay (ELISA) method. The recoveries in plasma and tissues were ranged from 82.3% to 107.0% and the intra- and inter-day precision values were below 15%. The developed cFLISA has been successfully applied to the measurement of the concentrations of RUS in biological samples of rats, and showed great potential for the tissue distribution study of RUS. The cFLISA method may provide a valuable tool for the analysis of small molecules in biological samples and such an approach could be applied to other natural products.

## 1. Introduction

Ruscogenin (RUS), a steroidal sapogenin isolated from *Ruscusacu leatus* and *Ophiopogon japonicus*, possesses a variety of pharmacological activities, including anti-inflammatory [1,2], platelet aggregation inhibition [3,4], pulmonary hypertension lowering [5,6], acute lung injury alleviating [7], liver injury attenuating [8] and other effects. Like many other steroidal saponins, there are no chromophoric groups in the structure of RUS, which leads to a lack of ultraviolet (UV) absorption. This factor inherently restricts the potential application of detection techniques involving UV to detect such kinds of steroidal saponins [9,10]. Although there is a large amount of research about the pharmacological functions and mechanism of RUS concerning cardiovascular and cerebrovascular diseases [11,12], the tissue distribution remains unknown. Nowadays, the analytical methods used to analyze RUS including HPLC-ELSD [13] and UPLC-MS/MS [14]. However, these methods are inconvenient due to their tedious sample pretreatment, large consumption of extraction solvents, time-consumption and expensive instruments. In addition to instrumental analysis, an indirect competitive ELISA for RUS was developed. Although this method is uniquely positioned as a simple and quick process compared with the instrumental analysis methods, it still required laborious and time-consuming incubation and washing steps. Additionally, enzyme-based labels suffer from instability due to denaturation and degradation. Therefore, it is necessary to establish a superior method to analyze RUS.

FLISA, is similar in principle to ELISA except that organic dyes take the place of enzymes. In the FLISA method, time- and cost-consuming secondary antibody reactions and subsequent enzyme-substrate reactions can be avoided, which obviously shortens the measurement time. However, there are still some limitations for the traditional FLISA based on the organic dyes such as low photoluminescence quantum yield and poor stability. Therefore, it is essential to search for new luminescent materials to replace the organic dyes to improve the stability of FLISA. QDs are semiconductor fluorescent nanoparticles which have many special properties such as high quantum yields, narrow and symmetric emission spectrum, broad excitation spectrum, large effective Stokes shifts and high photostability which makes them ideal for biological imaging and analysis [15,16,17]. In order to improve the sensitivity and selectivity, QDs are usually coupled with antibodies [16,18,19], which make it possible to complete the test time within one step. Nowadays, FLISA tests based on QDs and antibodies have been widely applied to the detection of low molecular weight analytes such as antibiotics, pesticides, hormones, toxins and so on and the LOD can be as low as pg level [20,21,22,23].

To overcome the problems existing in the detection of RUS, in the work reported here we combined a fluoroimmunoassay and QDs and established a novel cFLISA method to determine RUS with monoclonal antibodies conjugated with CdSe/ZnS quantum dots. This method was successfully applied to study the tissue distribution of RUS in Sprague-Dawley (SD) rats.

## 2. Results and Discussion 

### 2.1. The Conjugation

Conjugation is one of the key step in the establishment of the FLISA method. Therefore, it is of vital importance to choose a proper conjugation procedure. Nowadays, many conjugation techniques have been developed [16] such as direct conjugation using active esters, direct conjugation with 4-(*N*-maleimidomethyl)cyclohexanecarboxylic acid *N*-hydrosuccinimide ester (SMCC), indirect conjugation using avidin as bridge protein and biotinylated antibodies, and indirect conjugation using modified protein G. As one of the first employed conjugation procedures, the active esters method based on the reagents of EDC and NHS has unique advantages such as being simple, cheap, requiring common reagents, and is frequently performed in an one-pot reaction. The method has been widely employed by many researchers to conjugate QDs and antibodies, such as QDs to specific antibodies against morphine [24], quinoxaline-2-carboxylic acid [25], methyl-3-quinoxaline-2-carboxylic acid [25], ochratoxin A [26,27] and so on. Based on the above, in this paper, we choose the active esters method to conjugate the mAb and QDs.

**Figure 1:**
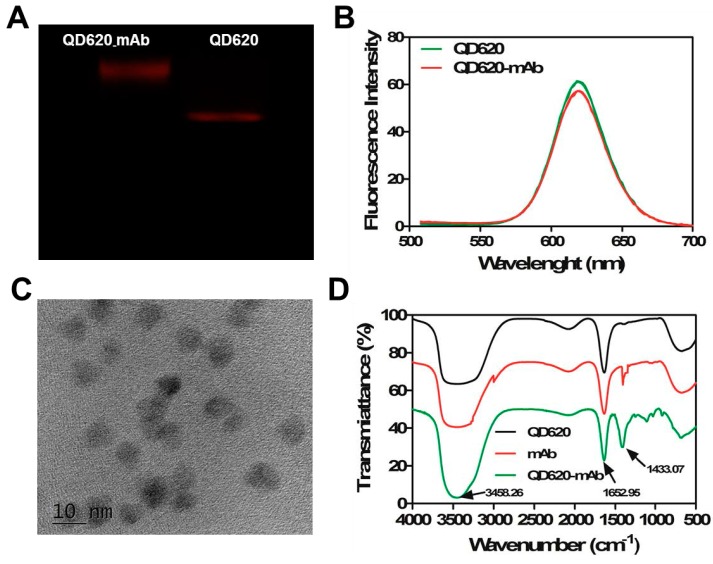
The conjugation of QD_620_ and monoclonal antibody anti-RUS. (**A**) the gel electrophoretic mobility of QD_620_ before and after conjugation; (**B**) the fluorescence spectra of QD_620_ before and after conjugation (The excitation wavelength was set at 400 nm); (**C**) the TEM images of QD_620_-mAbs; (**D**) the FT-IR spectra of QD_620_, mAbs, and QD_620_-mAbs.

The carboxylate groups on the surface of CdSe/ZnS QDs can be conjugated with the amino groups of anti-RUS monoclonal antibodies by the EDC coupling method. As shown in Figure 1, the agarose gel electrophoresis image preliminarily verified that mAb was successfully linked to the surface of QD_620_ because of the less migration of QD_620_-mAbs compared with the original QD_620_ (Figure 1A). In addition, the FTIR spectra of QD_620_-mAb and mAb showed characteristic amide bond peaks at 1652.95 cm (C=O amide I) and 1433.07 cm (N–C=O amide II), whereas QD_620_ just shows the C=O stretch that can be attributed to the modified carboxyl group in the absence of the amide II signal (Figure 1D). The presence of amide linkages on the QD_620_-mAbs demonstrated the successful covalent coupling between anti-RUS mAb and QD_620_. The fluorescence spectra of conjugates also had a max emission wavelength of 620 nm (λ_ex_ = 400 nm), which was the same as CdSe/ZnS QDs, indicated that the excellent optical properties of QDs coupled with mAbs were still being remained (Figure 1B). The fluorescence intensity of mAb-QDs was slightly weaker than CdSe/ZnS QDs due to two aspects, the loss of QDs and the fluorescent quenching during the course of reaction [28]. The TEM image (Figure 1C) showed that the QD_620_-mAb conjugates are spherical crystals, uniform in size (average diameter about 5 nm), and uniformly dispersed without aggregation.

### 2.2. Binding Affinity of the Conjugated mAb

**Figure 2:**
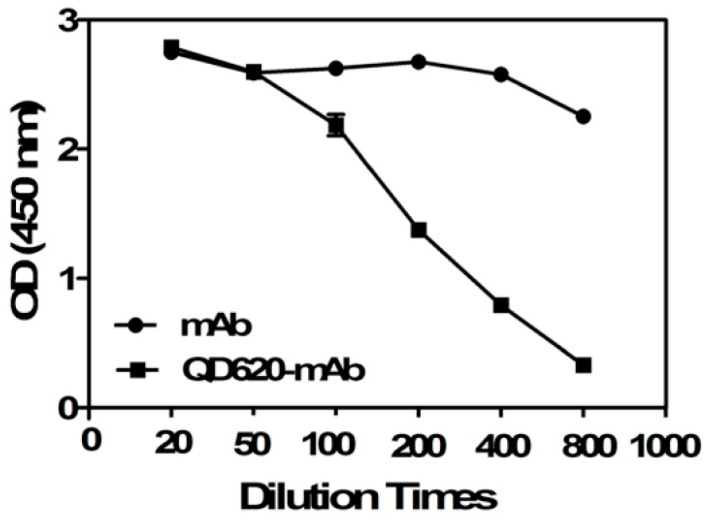
Comparison of the absorbance at 450 nm of mAb and QD620-mAb at different dilution times.

For the reason that orientation of the conjugation by EDC and NHS is not controlled, the reaction of the QD with the paratope of the antibody may occur, leading to poor antigen binding. To detect the biological activity of the mAb before and after QDs coupling, the indirect ELISA technique was conducted. In order to keep the recognition efficiency of the mAb during the conjugation process with the QDs, an excess amount of antibody was used. The results are shown in Figure 2.

When diluted 400 times, the OD_450_ value of the conjugates decreased from 2.78 to 0.80 while that of the mAb decreased from 2.79 to 2.25, which indicated that QD_620_-mAbs still retained bioactivity to interact with their counterparts. On the other hand, the biological activity of the mAbs were somewhat decreased after conjugation since the unbounded mAb were removed by centrifugation during the conjugation process [18]. In addition, the non-oriented reaction between mAb and QDs may decrease the biological activity of antibody because the QDs occupy some of the antigen-binding sites of the mAb. However, the QDs conjugated mAb still had ideal binding affinity and could be used in the subsequent FLISA assay.

### 2.3. FLISA Method

#### 2.3.1. The Calibration Curve

**Figure 3:**
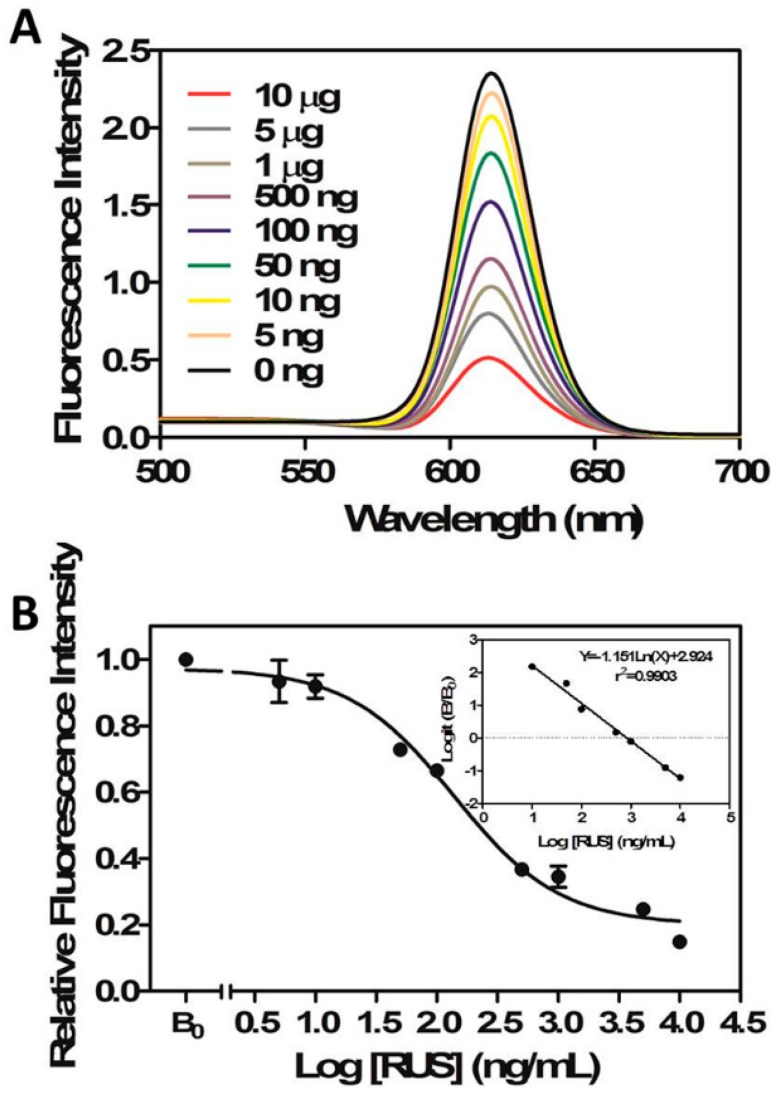
Fluorescence spectra of the FLISA based on QD-mAbs in the presence of various concentrations of RUS (0–10 μg/mL) under optimal experimental conditions (**A**); Standard curve for RUS (**B**). Each point represents the mean ± SD from three determinations in FLISA (excitation wavelength: 400 nm, emission wavelength: 620 nm).

The results are shown in Figure 3, and the calibration curve was then established with a perfect linear relationship (r^2^ = 0.9903) between inhibition efficiency and logarithm of RUS concentration when the concentration varied from 0.1 to 1000 ng/mL. Hereinto, the IC_50_ value and LOD were 9.6 ng/mL and 0.016 ng/mL, respectively.

#### 2.3.2. The Comparison of the FLISA and ELISA

**Figure 4:**
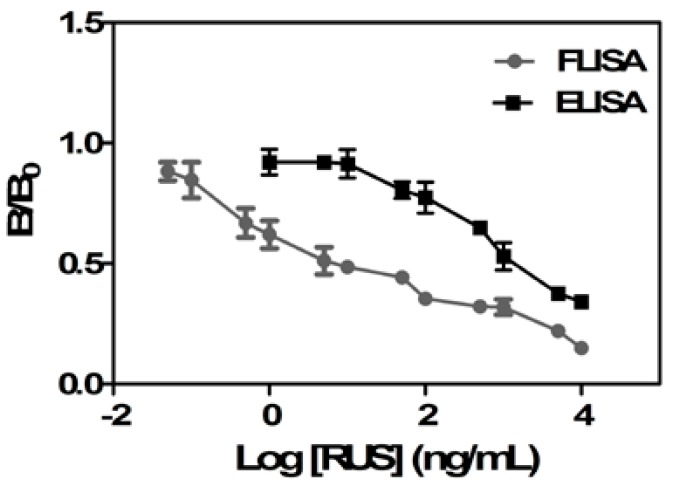
The inhibition curves for the determination of RUS by indirect competitive ELISA and FLISA.

The FLISA method is superior to the ELISA one due to the simple steps and short time. In addition to this, the IC_50_ and LOD of the FLISA assay were much lower than those of the ELISA method according to previous studies [26,29]. In this study, we compared the linear range, IC_50_ and LOD of the FLISA and ELISA methods. As shown in Figure 4, the FLISA method has a broader linear range. The IC_50_ value and LOD of ELISA method were 433.4 ng/mL and 5.4 ng/mL, respectively, which are much higher than those of the FLISA method (Table 1). In conclusion, the FLISA based on the QD-mAb exhibited higher sensitivity than the competitive ELISA based on the RUS mAb.

#### 2.3.3. The Cross Reactivity

**Figure 5:**
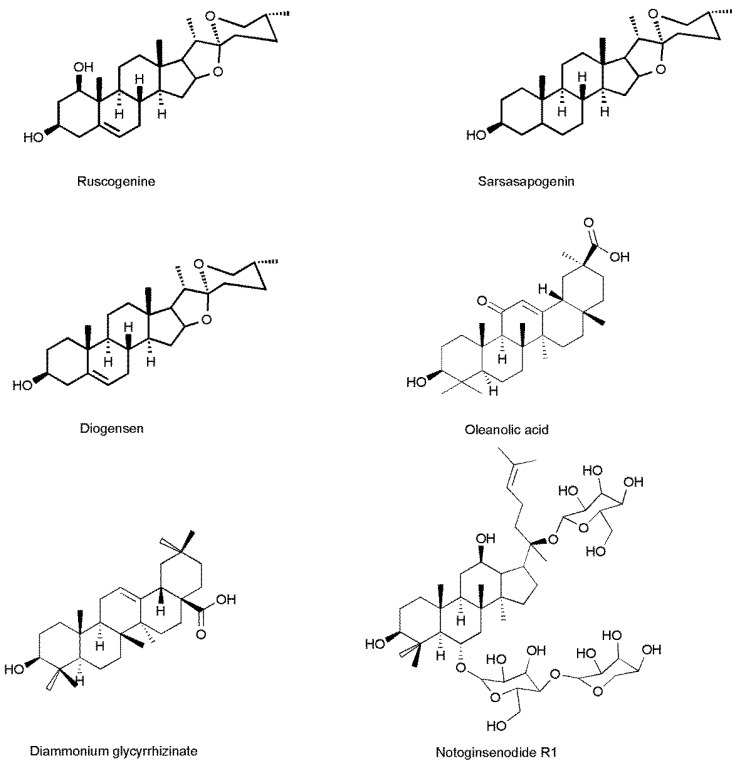
The structure of RUS and the analogues of RUS.

The cross reactivity of several analogs of RUS were determined and the results shown in Table 2. Diammonium glycyrrhizinate, sarsasapogenin, notoginsenoside R1 and oleanolic acid which have different skeletons (Figure 5) showed low cross-reactivity (lower than 0.1%). Diosgenin, just lacking one hydroxyl at the C-1 position in the skeleton compared with RUS only showed 22.3% cross-reactivity. These results indicated that RUS mAb had good specificity to the analogues in FLISA.

#### 2.3.4. The Recovery, Precision, Accuracy in Plasma and Tissues of Rats

To reduce the matrix effects, the biological samples were diluted and the calibration curves were obtained by adding the standard substances into the samples. The concentration range of all calibration curves of RUS in plasma and tissues of rats were from 0.05 ng/mL to 5000 ng/mL and almost all the samples had good linear relationship with r^2^ greater than 0.99 except the samples of liver and kidney (Table 3). Recovery in plasma and tissues were investigated at the three concentrations of RUS (1500 ng, 100 ng, 10 ng) and the results are summarized in Table 4. The recoveries in plasma and tissue samples ranged from 82.26% to 106.95% with RSD ≤ 13.0%. The intra- and inter-day precision and accuracy data of RUS are presented in Table 5. The precision was expressed as RSD% and accuracy expressed as RE%. These results demonstrated that the precision and accuracy values were within an acceptance range (<15%). The results showed that the FLISA method based on QD-mAb had good linear relations, higher precision and accuracy, and satisfactory recovery, which indicated that the method could be applied to the determination of RUS in biological samples.

### 2.4. RUS Distribution Study 

**Figure 6:**
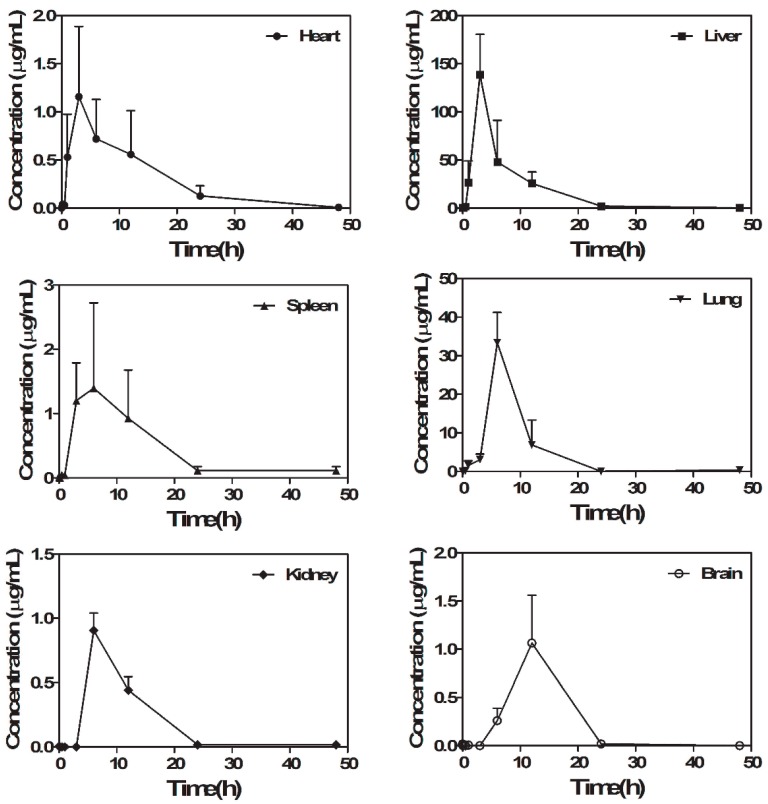

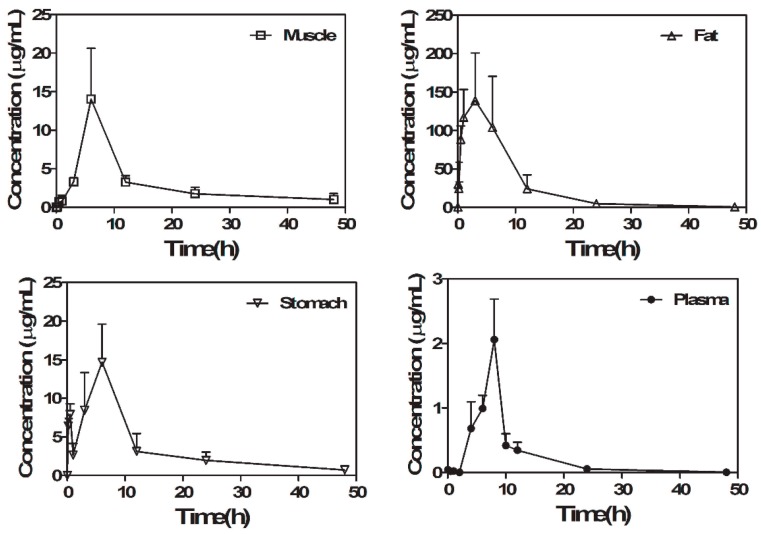
Concentration-time curves of RUS in rats tissues after oral administration (*n* = 6).

The FLISA method was successfully applied to investigate the tissue distribution of RUS and the study was determined at 0, 0.083, 0.167, 0.5, 1, 3, 6, 12, 24 and 48 h in brain, heart, kidney, liver, lung, spleen, stomach and muscle of SD rats and the oral administration of RUS was at a dose of 100 mg/kg. The results are shown in Figure 6. The RUS was rapidly and widely distributed in various tissues. The concentration of RUS was high in the liver, lung and fat, while obviously low in the heart, brain, kidney and spleen. The phenomenon indicated that extensive metabolism probably happened in the liver and the compound may accumulate in fat. The high concentration of RUS in the lung may suggest the potential target related to the pulmonary disease [5,6]. The low concentration in brain may indicate that the RUS can hardly cross through the blood brain barrier. The result also indicated that the RUS almost completely eliminated after 24 h in all the tissues.

## 3. Materials and Methods

### 3.1. Reagents

Carboxylic acid-modified CdSe/ZnS QDs with a maximum emission wavelength at 620 nm (QD_620_) was purchased from Wuhan Jiayuan Quantum Dots Co., Ltd. (Wuhan, China). 1-Ethyl-3-(3-dimethylaminopropyl) carbodiimide (EDC), *N*-hydroxysulfosuccinimide (sulfo-NHS), glucosamine hydrochloride (glu-NH_2_), bovine serum albumin (BSA), ovalbumin (OVA) were purchased from Sigma-Aldrich Chemical Co. (St. Louis, MO, USA). Agarose gel was from BIOWEST (Logan, UT, USA). RUS was isolated from the tubers of *Ophiopogon japonicus* by successive chromatographic steps and the purity was 99.6% by HPLC-ELSD. Diammonium glycyrrhizinate, sarsasapogenin, notoginsenoside R1, oleanolic acid and diosgenin with purities of 99.0% were purchased from the China Pharmaceutical Biological Products Analysis Institute (Beijing, China). The anti-RUS mAbs was derived from a hybridoma cell line which secreted stable monoclonal antibodies against RUS and purified by a Protein G affinity column (GE, Stockholm, Sweden) [30,31]. All the reagents were analytical grade.

### 3.2. Apparatus

The fluorescence spectra of QDs were measured by a Varioskan Flash spectrometer (Thermo, Waltham, MA, USA). Absorbance was measured by an Epoch microplate reader (Biotech). The agarose gel images were obtained by gel imaging analysis system (Bio-Rad, Hercules, CA, USA) and the Fourier transform infrared (FTIR) spectra were recorded by a Nicolet Impactor instrument (Thermo). Particle size was determined by transmission electron microscopy (TEM, JEM-1010, JEOL, Tokyo, Japan). Opaque white and the transparent polystyrene 96-well microtiter plates were purchased from Costar (Corning, NY, USA). Deionized water was prepared by a Milli-Q water purification system (Millipore, Billerica, MA, USA).

### 3.3. Animals

SD rats (200 ± 10 g, male) were obtained from Shanghai Jiesijie Laboratory Animal Co., Ltd (Shanghai, China, certificate No. SCXK 2015-0016). Animals were housed in a temperature-controlled environment with a 12-h-light-dark cycle and allowed free access to food and water. Prior to experiments, animals were randomized into different experimental groups and the indices were measured blindly. All procedures for animals were performed in accordance with the National Institutes of Health Guide for the Care and Use of Laboratory Animals and related regulations of China Pharmaceutical University.

### 3.4. Conjugation of QDs with Anti-RUS Monoclonal Antibody

The anti-RUS-mAb was coupled with QD_620_ through the method as previously described with some modifications [32,33]. Briefly, 10 μg EDC, 2.2 μg sulfo-NHS, and 0.1 nmol QD_620_ were dissolved in 0.5 mL borate buffer (pH 5.0; 0.05 M) and the mixture were reacted at room temperature for 30 min with gentle shaking. Then the solution pH was adjusted to 7.4, and the anti-RUS-mAb was added to the ester-activated QD_620_ followed by incubation at room temperature for 1 h. To block the excess carboxyl sites on the QD surface, the solution was mixed with 1000-fold molar of glu-NH_2_ and incubation for another 1 h. Finally, the mixture was added by glu-NH_2_ to the concentration of 2% (*w*/*v*), the pH was adjusted to 4.5. The products were separated by centrifugation at 20,000× *g* for 1 h at 4 °C and the precipitates were dispersed with borate buffer (pH 8.0; 0.05 M) and stored at 4 °C prior to use.

### 3.5. Identification of the Conjugates

The fluorescence spectra of QDs and conjugates were measured by the Varioskan Flash instrument with the excitation wavelength of 400 nm, an excitation slit of 2 nm, and the emission fluorescence intensity between 450 nm and 700 nm were recorded with an emission slit of 2 nm.

The QD-conjugates were further confirmed with agarose gel electrophoresis. Briefly, 0.25 g of agarose was added to 25 mL of Tris-acetate-EDTA buffer (TAE, pH 8.0) and heated until completely melted and then cooled down at room temperature in a gel tray with a 1.0 mm 11-well comb in forming the 1% (*w*/*v*) gel. For each well, 10 μL of the QDs/QD-conjugates samples were added in comb holes. The electrophoresis condition was 110 V, 80 mA for 30 min. The gel then imaged with 0.4 s exposure using the UV gel imaging system (BioRad). The FTIR were recorded within the range of 4000–400 cm^−1^. Particle size and morphology were determined by TEM.

### 3.6. Binding Affinity of the Conjugated mAb

To test the biological activity of the mAb before and after QDs coupling, the indirect ELISA technique was applied. The procedure was as follows [29]: briefly, transparent polyethylene plates were coated with RUS-OVA (10 μg/mL, 100 μL/well) which was dissolved in carbonate-bicarbonate buffer (pH 9.6; 0.05 M) and incubated overnight. Then the plates were washed three times with PBST (PBS with 0.05% Tween-20, *v*/*v*, 0.01 M; pH 7.4) and blocked with 0.5% gelatin in PBST (150 μL/well) for 1 h at 37 °C. The QD-mAbs was diluted into several series by PBST and added to the wells (100 μL/well). After incubation at 37 °C for 2 h and three washes, the plate was added by goat anti-mouse IgG-HRP (1:1000 in PBST, 100 μL/well) and incubated for 1 h at 37 °C. After three washes, 100 μL/well of TMB solution (1 mg TMB dissolved in 1 mL DMSO and mixed with 9 mL citrate buffer and 10 μL H_2_O_2_) was added and incubated for 20 min. Lastly, 2 M H_2_SO_4_ (50 μL/well) was added to cease the color development and the absorbance was measured with a microplate reader (Thermo) at 450 nm.

### 3.7. FLISA Procedure and Assessment of Method

#### 3.7.1. The FLISA Procedure

**Figure 7:**
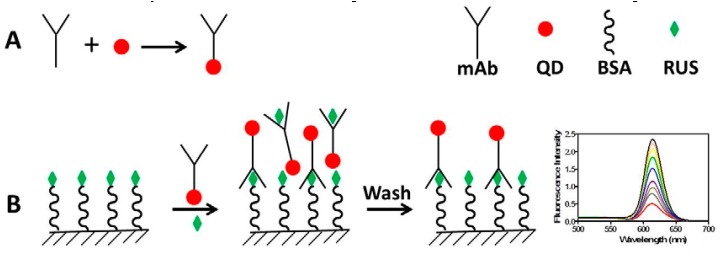
Schematic diagram of cFLISA.

The FLISA method was as shown in Figure 7. The procedure was performed according to relevant references with some modifications [34]. Briefly, opaque white microtiter plates were coated with 100 μL RUS-BSA (10 μg/mL) in carbonate-bicarbonate buffer (pH 9.6; 0.05 M) and incubated at 4 °C for overnight. After three washes, the plate was blocked with 0.5% gelatin in PBST with 150 μL/well and incubated for 1 h at 37 °C. Then, 50 μL of standard serial dilutions of RUS were added into the plate followed by the 50 μL of diluted mAb-QD conjugates (1:100) and incubated for 2 h at 37 °C. After three washes, the plate was added 100 μL of PBS (pH 7.4; 0.01 M) into each well and recorded the fluorescence intensity with excitation wavelength of 400 nm and emission wavelength of 620 nm.

#### 3.7.2. Competition Curves

Different concentrations of RUS ranging from 0 ng/mL to 10 μg/mL were dissolved in PBST which contained 10% methanol as the standard solution and determined by the method described above. Competitive curves were generated by plotting the logit of inhibition values versus the logarithm of RUS concentration. In the curves, IC_50_ values were determined as the concentration at which 50% inhibition by binding the antibody to the coating antigen.

#### 3.7.3. The Comparison of the FLISA and ELISA

The comparison of the FLISA and ELISA was conducted by testing the same concentrations of RUS ranging from 0 ng/mL to 10 μg/mL with FLISA and ELISA methods, respectively. Then the competitive inhibition curves were plotted.

#### 3.7.4. Cross Reaction of the Analogues of RUS

The cross reactivity was determined as the IC_50_ values of analogues of RUS which required for 50% inhibition of the binding of the antibodies to the solid-phase antigen, as compared to RUS itself (100%), which responded to the following formula:

Cross reactivity = [IC_50_ (analogues)/IC_50_ (RUS)] × 100%.

#### 3.7.5. The Method Assessment

The study of the validation and reliability of the FLISA method including several parameters such as recovery of RUS spiked in the biological samples, intra- and inter-day assay precision and LOD of the assays.

##### Recovery Analysis

Biological samples of blood, brain, heart, kidney, liver, lung, spleen, fat, spinal cord and muscle were collected from six blank SD rats. The blood samples were centrifuged at 3000 rpm for 15 min at 4 °C to obtain the plasma. All tissues sample were thoroughly rinsed in ice cold saline to eliminate blood and dried with filter paper. An accurately weighted amount of each tissue sample was homogenized in PBS (0.01 M, pH 7.4, 3:1, *w*/*v*). The blank samples were spiked with RUS to achieve concentrations of 1500, 100 and 10 ng/mL. Then methanol (4:1, *w*/*v*) was added into and vortex-mixed for 30 s. The mixture was centrifuged at 12,000 rpm for 15 min at 4 °C and the supernatant was transferred to another tube. The supernatant was diluted 8 times to yield sample solutions in 10% methanol. The recovery was calculated as the percentage ratio of the determined concentration and added concentration.

##### The Intra- and Inter-Day Precision and Accuracy

The intra-day assay was carried out by the measurement of the spiked samples three times within a day. The inter-day assay measured the spiked samples during three continuous days. The precision was showed as relative standard deviation (RSD) and the accuracy was as the relative error (RE). The value of RE was calculated as the following function:

RE = (x − μ)/x × 100%

In which x was the added value and μ was the detection value.

##### The LOD

The LOD was the mean value of six blank samples plus three times standard deviations on the standard curve.

### 3.8. The Tissue Distribution Study

For the tissue distribution study, 60 SD rats randomly were divided into 10 groups (n = 6). Samples of the brain, heart, kidney, liver, lung, spleen, stomach and muscle were collected at designated times (0, 0.083, 0.167, 0.5, 1, 3, 6, 12, 24 and 48 h) following euthanasia by CO_2_. Every tissue sample was thoroughly rinsed in ice cold saline to eliminate blood and other content and blotted dry with filter paper. An accurately weighted amount of each tissue samples were homogenized in PBS (1:3, *w*/*v*; 0.01 M; pH 7.4). The plasma and tissue homogenates were added into methanol (1:4, *v*/*v*) and vortex-mixed for 30 s. Then the mixture was centrifuged at 12,000 rpm for 15 min at 4 °C and the supernatant was diluted eight times to yield sample solutions in 10% methanol [34].

### 3.9. Statistical Analysis

All experimental data were processed by Microsoft Excel 2010 and Origin Pro 8.0 software. All data was obtained from three independent experiments and presented as the mean ± SD.

## 4. Conclusions

A sensitive and rapid FLISA method for the quantitative analysis of RUS was developed and successfully applied for the analysis of plasma and tissue samples in this study. The anti-RUS mAb could be successfully conjugated to CdSe/ZnS QDs by the catalysis of EDC. The conjugates retained biological activity without changing the optical characteristics. The FLISA method showed lower IC_50_ and LOD compared with traditional ELISA. The obtained tissue distribution data showed that RUS could be absorbed and distributed quickly in in brain, heart, kidney, liver, lung, spleen, stomach and muscle with a peak concentration of 138.5 μg/mL in liver and minimum concentration of 1.06 μg/mL in the brain.

The tissue distribution study indicated that the mainly metabolization pathway of RUS was in liver and build up in fat, while it could hardly cross the blood-brain barrier. In conclusion, a new FLISA method based on the QD-mAb made the fast and sensitive detection of RUS come true. Furthermore, this study provided a new method for the study of tissue distribution of RUS and this method could be applied to the study of other natural products.

## Figures and Tables

**Table 1:** Comparison of several parameters of the competitive FLISA and ELISA for RU.

	LOD (ng/mL)	IC_50_ (ng/mL)	Linear Range (ng/mL)
ELISA	5.43	433.4	1–5000
FLISA	0.016	9.59	0.1–10,000

**Table 2:** Cross reactivities of mAb against various compounds directed by FLISA.

Compounds	Cross Reactivities (%)
Sarsasapogenin	<0.1
Diammonium glycyrrhizinate	<0.1
Diosgenin	22.39
NotoginsenosideR1	<0.1
Oleanolic acid	<0.1
Ruscogenin	100

**Table 3:** Calibration curves for RUS in different biological samples.

Biological Sample	Calibration Curve	R^2^	Concentration Range (ng/mL)
Heart	*Y* = −0.7633*X* + 1.817	0.993	0.05–5000
Liver	*Y* = −1.154*X* + 3.859	0.9862	0.05–5000
Spleen	*Y* = −1.638*X* + 5.298	0.9902	0.05–5000
Lung	*Y* = −1.749*X* + 6.163	0.9906	0.05–5000
Kidney	*Y* = −0.9799*X* + 3.623	0.9887	0.05–5000
Brain	*Y* = −0.8733*X* + 3.184	0.9957	0.05–5000
Muscle	*Y* = −0.8592*X* + 3.589	0.9928	0.05–5000
Fat	*Y* = −1.241*X* + 3.997	0.9871	0.05–5000
Stomach	*Y* = −1.201*X* + 5.095	0.9917	0.05–5000
Plasma	*Y* = −1.154*X* + 3.804	0.9952	0.05–5000

**Table 4:** Recovery of RUS in rat plasma and tissue homogenates (n = 6).

Samples	Spiked (ng/mL)	Recovery	
		Mean (%)	RSD (%)
Heart	1500	85.6	4.94
	100	95.87	4.59
	10	91.22	6.04
Liver	1500	86.98	3.32
	100	92.15	6.38
	10	91.9	6.17
spleen	1500	87.67	3.16
	100	87.53	6.89
	10	93.34	7.93
Lung	1500	93.35	5.16
	100	87.55	8.54
	10	93.59	4.45
kidey	1500	87.38	3.18
	100	95.07	3.63
	10	91.45	6.32
Brain	1500	94.19	5.05
	100	91.39	6.12
	10	95.36	4.16
Muscle	1500	92.59	5.26
	100	85.39	3.41
	10	88.47	8.45
Fat	1500	98.83	4.13
	100	82.26	9.27
	10	91.26	8.76
Stomach	1500	91.78	7.02
	100	95.46	4.3
	10	100.22	0.87
Plasma	1500	88.77	2.38
	100	106.95	13.99
	10	94.75	9.29

**Table 5:** Precision and accuracy for determination of RUS in rat plasma and tissues by FLISA Method (n = 6).

Matrix	Accuracy RE (%)	Intra-Day RSD (%)	Inter-Day RSD (%)
Heart	−14.4	4.94	5.16
	−4.13	4.59	7.49
	−8.78	6.04	1.87
Liver	−13.02	3.32	3.48
	−7.85	6.38	1.07
	−8.1	6.17	2.5
Spleen	−12.33	3.16	1.4
	−12.47	6.89	6.49
	−6.66	7.93	8.9
Lung	−6.65	5.16	3.68
	−12.45	8.54	5.12
	−6.41	4.45	4.19
Kidney	−12.63	3.18	2.59
	−4.93	3.63	2.1
	−8.55	6.32	2.67
Brain	−5.81	5.05	4.12
	−8.61	6.12	1.83
	−4.64	4.16	6.41
Muscle	−7.41	5.26	6.72
	−14.61	3.41	3.92
	−11.53	8.45	6.98
Fat	−1.17	4.13	6
	−7.74	9.27	4.58
	−8.74	8.78	2.38
Stomach	−8.22	7.02	3.95
	−4.54	4.3	2.32
	0.22	0.89	9.33
Plasma	−11.23	2.39	6.5
	6.95	13.99	5.11
	−5.25	9.28	7.88

## References

[1] Cao G., Jiang N., Hu Y., Zhang Y., Wang G., Yin M., Ma X., Zhou K., Qi J., Yu B. (2016). Ruscogenin Attenuates Cerebral Ischemia-Induced Blood-Brain Barrier Dysfunction by Suppressing TXNIP/NLRP3 Inflammasome Activation and the MAPK Pathway. Int. J. Mol. Sci..

[2] Liu H., Zheng Y.F., Li C.Y., Zheng Y.Y., Wang D.Q., Wu Z., Huang L., Wang Y.G., Li P.B., Peng W. (2015). Discovery of Anti-inflammatory Ingredients in Chinese Herbal Formula Kouyanqing Granule based on Relevance Analysis between Chemical Characters and Biological Effects. Sci. Rep..

[3] Yu B.-Y. (2007). Exploration on the Modern Research Methodology of Traditional Chinese Medicine, Based on the Systemic Research of Radix Ophiopogonis. Chin. J. Nat. Med..

[4] Kou J., Tian Y., Tang Y., Yan J., Yu B. (2006). Antithrombotic Activities of Aqueous Extract from Radix Ophiopogon japonicus and Its Two Constituents. Biol. Pharm. Bull..

[5] Bi L.Q., Zhu R., Kong H., Wu S.L., Li N., Zuo X.R., Zhou S.M., Kou J.P., Yu B.Y., Wang H. (2013). Ruscogenin attenuates monocrotaline-induced pulmonary hypertension in rats. Int. Immunopharmacol..

[6] Zhu R., Bi L., Kong H., Xie W., Hong Y., Wang H. (2015). Ruscogenin exerts beneficial effects on monocrotaline-induced pulmonary hypertension by inhibiting NF-κB expression. Int. J. Clin. Exp. Pathol..

[7] Sun Q., Chen L., Gao M., Jiang W., Shao F., Li J., Wang J., Kou J., Yu B. (2012). Ruscogenin inhibits lipopolysaccharide-induced acute lung injury in mice: involvement of tissue factor, inducible NO synthase and nuclear factor (NF)-kappaB. Int. Immunopharmacol..

[8] Feihua W., Jingsong C., Jieyun J., Yu B., Xu Q. (2000). Ruscogenin glycoside (Lm-3) isolated fromLiriope muscariimproves liver injury by dysfunctioning liver-infiltrating lymphocytes. J. Pharm. Pharmacol..

[9] Liu N., Wen X., Liu J., Liang M., Zeng H., Lin Y., Yu B. (2006). Determination of ruscogenin in crude Chinese medicines and biological samples by immunoassay. Anal. Bioanal. Chem..

[10] Güvenç A., Şatır E., Coşkun M. (2007). Determination of Ruscogenin in Turkish Ruscus L. Species by UPLC. Chromatographia.

[11] Guan T., Liu Q., Qian Y., Yang H., Kong J., Kou J., Yu B. (2013). Ruscogenin reduces cerebral ischemic injury via NF-kappaB-mediated inflammatory pathway in the mouse model of experimental stroke. Eur. J. Pharmacol..

[12] Lin Y.N., Jia R., Liu Y.H., Gao Y., Wang L.L., Kou J.P., Yu B.Y. (2015). Ruscogenin suppresses mouse neutrophil activation: Involvement of protein kinase A pathway. J. Steroid Biochem. Mol. Biol..

[13] Liu C.-H., Li M.L., Feng Y.-Q., Hu Y.-J., Yu B.-Y., Jin Q. (2016). Determination of Ruscogenin in Ophiopogonis Radix by High-performance Liquid Chromatography-evaporative Light Scattering Detector Coupled with Hierarchical Clustering Analysis. Pharmacogn. Mag..

[14] Ji P.Y., Li Z.W., Yang Q., Wu R. (2015). Rapid determination of ruscogenin in rat plasma with application to pharmacokinetic study. J. Chromatogr. B Anal. Technol. Biomed. Life Sci..

[15] Rosenthal S.J., Chang J.C., Kovtun O., McBride J.R., Tomlinson I.D. (2011). Biocompatible quantum dots for biological applications. Chem. Biol..

[16] Esteve-Turrillas F.A., Abad-Fuentes A. (2013). Applications of quantum dots as probes in immunosensing of small-sized analytes. Biosens. Bioelectron..

[17] Murphy C.J. (2002). Optical sensing with quantum dots. Anal. Chem..

[18] Zhu K., Li J., Wang Z., Jiang H., Beier R.C., Xu F., Shen J., Ding S. (2011). Simultaneous detection of multiple chemical residues in milk using broad-specificity antibodies in a hybrid immunosorbent assay. Biosens. Bioelectron..

[19] Ding S., Chen J., Jiang H., He J., Shi W., Zhao W., Shen J. (2006). Application of Quantum Dot—Antibody Conjugates for Detection of Sulfamethazine Residue in Chicken Muscle Tissue. J. Agric. Food Chem..

[20] Shen J., Xu F., Jiang H., Wang Z., Tong J., Guo P., Ding S. (2007). Characterization and application of quantum dot nanocrystal-monoclonal antibody conjugates for the determination of sulfamethazine in milk by fluoroimmunoassay. Anal. Bioanal. Chem..

[21] Fernandez-Arguelles M.T., Costa-Fernandez J.M., Pereiro R., Sanz-Medel A. (2008). Simple bio-conjugation of polymer-coated quantum dots with antibodies for fluorescence-based immunoassays. Analyst.

[22] Wang X., Tao G., Meng Y. (2009). A Novel CdSe/CdS Quantum Dot-based Competitive Fluoroimmunoassay for the Detection of Clenbuterol Residue in Pig Urine Using Megnetic Core/Shell Fe3o4/Au Nanoparticals as a Solid Carrier. Anal. Sci. Dec..

[23] Chen Y., Ren H.L., Liu N., Sai N., Liu X., Liu Z., Gao Z., Ning B. (2010). A fluoroimmunoassay based on quantum dot-streptavidin conjugate for the detection of chlorpyrifos. J. Agric. Food Chem..

[24] Zhang C., Han Y., Lin L., Deng N., Chen B., Liu Y. (2017). Development of Quantum Dots-Labeled Antibody Fluorescence Immunoassays for the Detection of Morphine. J. Agric. Food Chem..

[25] Le T., Zhu L., Yu H. (2016). Dual-label quantum dot-based immunoassay for simultaneous determination of Carbadox and Olaquindox metabolites in animal tissues. Food Chem..

[26] Yao J., Xing G., Han J., Sun Y., Wang F., Deng R., Hu X., Zhang G. (2017). Novel fluoroimmunoassays for detecting ochratoxin A using CdTe quantum dots. J. Biophotonics.

[27] Xiong S., Zhou Y., Huang X., Yu R., Lai W., Xiong Y. (2017). Ultrasensitive direct competitive FLISA using highly luminescent quantum dot beads for tuning affinity of competing antigens to antibodies. Anal. Chim. Acta.

[28] Medintz I.L., Clapp A.R., Mattoussi H., Goldman E.R., Fisher B., Mauro J.M. (2003). Self-assembled nanoscale biosensors based on quantum dot FRET donors. Nat. Mater..

[29] Zhang Z., Li Y., Li P., Zhang Q., Zhang W., Hu X., Ding X. (2014). Monoclonal antibody-quantum dots CdTe conjugate-based fluoroimmunoassay for the determination of aflatoxin B1 in peanuts. Food Chem..

[30] Xu Y., Liu J.-H., Wang J., Zhang J., Yu B.-Y. (2014). A monoclonal antibody-based competitive ELISA for the determination of ruscogenin in Chinese traditional medicines and biological samples. Chin. J. Natl. Med..

[31] Liu H.Y., Gao X. (2011). Engineering monovalent quantum dot-antibody bioconjugates with a hybrid gel system. Bioconj. Chem..

[32] Xu W., Xiong Y., Lai W., Xu Y., Li C., Xie M. (2014). A homogeneous immunosensor for AFB1 detection based on FRET between different-sized quantum dots. Biosens. Bioelectron..

[33] Yang A., Zheng Y., Long C., Chen H., Liu B., Li X., Yuan J., Cheng F. (2014). Fluorescent immunosorbent assay for the detection of alpha lactalbumin in dairy products with monoclonal antibody bioconjugated with CdSe/ZnS quantum dots. Food Chem..

[34] Lee N.A., Wang S., Allan R.D., Kennedy I.R. (2004). A Rapid Aflatoxin B1 ELISA: Development and Validation with Reduced Matrix Effects for Peanuts, Corn, Pistachio, and Soybeans. J. Agric. Food Chem..

